# The absence of formal work experience may affect the rate of cognitive decline in older adult women: findings from the health and retirement study

**DOI:** 10.3389/fgwh.2024.1458553

**Published:** 2024-09-16

**Authors:** Daya K. Grewal, Molly A. Patapoff, Victoria Liou-Johnson, Maheen M. Adamson, Dylan J. Jester

**Affiliations:** ^1^ Women’s Operational Military Exposure Network Center of Excellence (WOMEN CoE), VA Palo Alto Health Care System, Palo Alto, CA, United States; ^2^ Department of Psychology, Palo Alto University, Palo Alto, CA, United States; ^3^ Sam and Rose Stein Institute for Research on Aging, University of California San Diego, La Jolla, CA, United States; ^4^ Department of Psychiatry, University of California San Diego, La Jolla, CA, United States; ^5^ Rehabilitation Service, VA Palo Alto Health Care System, Palo Alto, CA, United States; ^6^ Clinical Excellence Research Center, Stanford University School of Medicine, Stanford, CA, United States; ^7^ Department of Neurosurgery, Stanford University School of Medicine, Stanford, CA, United States

**Keywords:** cognitive reserve, occupation, gender norms, socioeconomic factors, ethnic minority, aging

## Abstract

**Objective:**

This study investigated the relationship between years of employment and cognitive health among older non-Latinx Black, Latinx, and non-Latinx White women. We hypothesized that women who had never been formally employed (i.e., zero years of formal work experience) would exhibit a pronounced cognitive decline.

**Methods:**

Our study included 5,664 older adult women from the Health and Retirement Study (2010–2016) aged 65–101 (*M* = 75.41). Out of 5,664 participants, 850 identified as non-Latinx Black, 475 identified as Latinx, and 4,339 identified as non-Latinx White. Furthermore, 5,292 women indicated having a professional employment history of at least one year, whereas 372 women reported no formal work experience. The Telephone Interview for Cognitive Status-27 (TICS-27) was used to assess cognitive performance. Linear mixed effects models were conducted to assess whether employment history was associated with the rate of cognitive decline.

**Results:**

In all three racial and ethnic groups, lower age, higher education, greater number of years worked, fewer chronic conditions, and greater household income were associated with better cognitive performance at baseline (*p* < .05). Additionally, women who had not worked in any formal capacity had a lower baseline cognitive performance (*p *< .001) and a more extreme decline in cognitive performance over time (*p = .*04).

**Conclusion:**

In conclusion, we found that women without any formal work experience performed lower at baseline and experienced a steeper cognitive decline over time. These findings underscore the need to further explore the complex interrelationships between employment duration and cognitive trajectories, especially among older women and those from different racial and ethnic backgrounds.

## Introduction

Cognitive decline remains one of the world's most burdensome chronic health conditions. Rates of Alzheimer's disease and related dementias (ADRD) have risen in recent years (1), and disproportionately across marginalized racial and ethnic groups. Non-Latinx Black and Latinx older adults have an increased risk of receiving an ADRD diagnosis when compared to their non-Latinx White counterparts (2–4), and this higher rate of ADRD is largely due to the effects of systemic racism on health and educational, occupational, and social opportunities. Additionally, women have an increased risk of developing ADRD (5). This greater risk may be linked to several factors: women are more likely than men to live into older age (6), there are sex differences in ADRD neuropathology and changes in hormones during menopause may affect risk (1, 5), and gender discrimination in education and work opportunities may result in lower cognitive reserve (CR) (7, 8).

### Cognitive reserve

CR helps explain the discrepancies between neuropathology and clinical functioning. Individuals with high CR may not show symptoms of ADRD, despite significant brain atrophy and network disruption (9). CR has been linked to a 47% reduction in the risk of progressing to Mild Cognitive Impairment (MCI) or ADRD, independent of structural pathology and Alzheimer's biomarkers (10). CR enables alternative neuronal networks and cognitive strategies to maintain performance despite brain changes (11). However, operationalizing CR is complex due to varying definitions that influence the perceived risk of MCI or ADRD. Proxies like educational attainment, occupational complexity, and participation in intellectually enriching activities are often used as indirect measures of CR, assuming that higher levels indicate greater reserve (12). Another approach uses residual cognitive performance after adjusting for AD neuropathology to measure CR (10).

CR is significantly shaped by social determinants of health (SDoH), including education, occupational history, and socioeconomic status (SES). Education strengthens and creates neural networks (13–15) and serves as a proxy for higher SES. Those with higher educational attainment often secure better-paying jobs, leading to health-promoting behaviors, reduced social stressors, and a lower risk of cognitive decline (7, 16, 17). Occupation, as a core SES indicator, supports health maintenance by contributing to economic, cultural, or social capital (16, 18) and may directly influence cognitive outcomes (19).

### The role of occupational history on cognitive reserve

Adulthood is largely occupied with work-related activities that demand significant time and energy (20). Jobs involving monotonous, low-skill tasks (low job complexity) and limited autonomy (low job control) are associated with poorer cognitive performance (21). In contrast, roles requiring higher cognitive engagement and complex social interactions are linked to better cognitive performance (22–27), reduced risk of ADRD (28), and a slower rate of cognitive decline post-retirement (29, 30), and roles with high autonomy correlate with increased hippocampal volume and a slower reduction in this volume over time (31, 32). Additionally, the cognitive benefits of job complexity appear to be moderated by leisure activity, especially social activities (22).

### Racial discrimination's impact on occupation and cognitive reserve

Simons et al. (33) suggest a possible role of SES and discrimination on accelerated biological aging through the accrual of chronic illnesses. The impacts of racial discrimination and chronic stress due to socioeconomic hardship, substandard education quality, and neighborhood disadvantages are linked to cognitive impairment and an increased risk of ADRD (2–4). Differences in SDoH account for 39% of the non-Latinx Black- non-Latinx White disparity and 76% of the Latinx- non-Latinx White disparity in cognitive performance (34), with years worked explaining 6% and 10% of these disparities, respectively. However, the protective effects of higher education or occupational complexity on cognitive decline may not apply equally across non-Latinx Black, Latinx, and non-Latinx White adults (18, 35–38), in part due to poorer educational quality and limited opportunities for occupational mobility among marginalized groups (39).

### Gender norms impact on occupation and cognitive performance

Gender disparities shape how occupational history impacts cognitive performance in older adulthood (40, 41). Occupational roles, often limited by historical gender norms, significantly influence cognitive health in later life (8, 42). Women, especially those from older birth cohorts, were subject to lower expectations for educational attainment and occupational status, often confined to roles with less cognitive complexity that could impact the rate of cognitive decline years later (22, 41). This effect is even more pronounced in women of color, who have faced compounded challenges due to intersecting sexism and racism, further limiting their educational and occupational opportunities and, thus, influencing their cognitive health (22, 43).

This study examined the impact of formal work experience on cognitive decline in older women, focusing on non-Latinx Black, Latinx, and non-Latinx White women to understand how years of formal employment affected cognitive trajectories. We hypothesized that having greater formal employment history would affect the rate of change on the TICS-27 over time (i.e., the interaction of employment duration by time) and that this effect would differ among non-Latinx Black, Latinx, and non-Latinx White women. Furthermore, we hypothesized that women who had never been formally employed (i.e., zero years of formal work experience) would exhibit a pronounced rate of cognitive decline.

## Methods

### Participants and study design

This is a retrospective cohort study examining a sample of older adult women (>65 years) from the Health and Retirement Study (HRS). The present study utilizes longitudinal data from the following four consecutive assessments: wave 10 (2010), wave 11 (2012), wave 12 (2014), and wave 13 (2016). The HRS is a collaboration between the National Institute of Aging (U01AG009740) and the University of Michigan, started in 1992. Managed by the University of Michigan's Survey Research Center, the study has maintained response rates between 81.7% and 89.1% since inception (49).

Inclusion criteria were: (1) aged ≥ 65 years at wave 10 and (2) identified as non-Latinx Black, Hispanic/Latinx, or non-Latinx White. Participants that identified as “other,” or were missing race and ethnicity data were excluded. Outcome: The Telephone Interview for Cognitive Status-27 (TICS-27) was used to assess cognitive performance. The TICS-27 is a 27-item measure of global cognition, scored using the Langa-Weir composite scoring approach (44). This method allocates 20 points to short-term and long-term memory and 7 points to processing speed and executive functions, with total scores ranging from 0 to 27.

### Covariates

The following covariates were included to control for possible confounding contributors to cognitive decline: age (years), education (years), body mass index, annual household income (natural-logged U.S. dollars), and number of chronic health conditions, including high blood pressure, diabetes, cancer, lung disease, heart problems, stroke, psychological problems, and arthritis. All covariates were estimated at participants’ 2010 (wave 10) assessment which functioned as a baseline for our analyses.

### Statistical analysis

Linear mixed effects models (LMMs) were conducted to assess the impact of employment duration on the rate of cognitive decline (i.e., change in cognitive performance over time). In this multilevel LMM framework, cognitive performance at each wave (Level 1) was nested within each participant (Level 2) to determine how women performed over time. Models were stratified by race and ethnicity (non-Latinx Black, Latinx, and non-Latinx White). The main independent variable of interest was employment duration (number of years worked), and the main outcome variable of interest was performance on the TICS-27 score measured consecutively over four waves. The coefficient for employment duration describes the average change in cognitive performance at baseline for each additional year of formal work experience. The coefficient for time describes the average change in cognitive performance over each wave (in roughly 2-year increments). An interaction term of time by employment duration describes the impact of each additional year of formal work experience on the average rate of change in cognitive performance. LMMs included a random intercept to capture interindividual differences at baseline. A random slope capturing intraindividual differences could not be estimated due to low within-person variance over time (i.e., the rate of change was largely homogeneous).

An additional LMM using the full sample (all three race and ethnicity groups) was constructed to examine cognitive decline in women with some vs. no formal work experience. In this model, employment duration was dummy coded as a categorical variable (zero years worked vs. one or more years work). An interaction term of time by the categorical employment duration variable assessed the impact of having no formal work experience on the average rate of change in cognitive performance. Due to significant differences in education between the two employment duration groups (i.e., a large effect size difference), models with education and without education are presented. As a sensitivity analysis, all models were reconducted with the TICS-20 memory component subscore (10 points immediate recall; 10 points delayed recall).

## Results

The final sample included 5,664 women aged 65–101 (*M* = 75.41, *SD = *7.22), of which 850 identified as non-Latinx Black, 475 identified as Latinx, and 4,339 participants identified as non-Latinx White. 5,292 women (93%) indicated having a professional employment history of at least one year, whereas 372 women (7%) reported no formal work experience. See Table 1 for baseline demographic information stratified by race and ethnicity.

**Table 1:** Demographic characteristics by race and ethnicity group.SD, standard deviation; TICS-27, telephone interview for cognitive status–modified 27-item.

Variables	Full sample (*n* = 5,664)		Non-Latinx black (*n* = 850)		Latinx (*n* = 475)		Non-Latinx white (*n* = 4,339)	
	Mean	SD	Mean	SD	Mean	SD	Mean	SD
Age (years)	75.41	7.22	74.31	6.76	74.20	6.42	75.75	7.36
Education (years)	12.25	2.97	11.72	2.98	8.26	4.53	12.80	2.33
Employment duration (years worked)	29.78	16.66	32.86	15.89	21.36	17.27	30.09	16.45
Annual household income	$43,836.49	$68,247.68	$26,536.52	$27,846.32	$23,019.94	$28,136.51	$49,504.35	$75,524.32
Body mass index	27.63	6.12	29.90	6.83	28.80	6.19	27.06	5.85
Chronic health conditions	2.46	1.40	2.68	1.35	2.46	1.44	2.42	1.40
TICS-27 total score at baseline/wave 10	14.21	4.62	11.91	4.82	11.81	4.67	14.93	4.34
TICS-27 total score at wave 11	14.05	4.66	11.94	4.72	11.43	4.74	14.74	4.41
TICS-27 total score at wave 12	13.90	4.77	11.45	4.84	11.08	4.66	14.67	4.49
TICS-27 total score at wave 13	13.62	4.75	11.40	4.64	10.65	4.73	14.41	4.49
TICS-20 memory component subscore at baseline/wave 10	9.26	3.48	8.17	3.50	8.20	3.37	9.59	3.42
TICS-20 memory component subcore at wave 11	9.14	3.49	8.10	3.44	7.78	3.39	9.49	3.43
TICS-20 memory component subcore at wave 12	8.98	3.59	7.73	3.49	7.45	3.40	9.38	3.53
TICS-20 memory component subscore at wave 13	8.74	3.53	7.66	3.32	7.12	3.37	9.14	3.50

In all three racial and ethnic groups, lower age, higher education, greater number of years worked, fewer chronic conditions, and greater household income were significantly associated with higher TICS-27 scores (indicating better cognitive performance) at baseline (*p *< .05). In the non-Latinx Black and non-Latinx White groups, higher body mass index was also significantly associated with higher TICS-27 scores at baseline. No significant interactions were observed between duration of employment and slope of cognitive performance in any of the racial and ethnic groups (*p *> .05; Table 2). Removing educational attainment as a covariate from the race and ethnicity stratified analyses did not appreciably change the results.

**Table 2:** Linear mixed effects models assessing the association of employment duration, covariates, and cognitive decline assessed via TICS-27 total score stratified by race and ethnicity.

	Model 2 Non-Latinx black			Model 3 Latinx			Model 1 Non-Latinx white		
	B	CI	*p*	B	CI	*p*	B	CI	*p*
Age (years)	−.201	(−.237, −.165)	<.001	.298	(−.236. −.134)	<.001	−.215	(−.229, −.200)	<.001
Education (years)	.588	(.509, .668)	<.001	−.185	(.222, .373)	<.001	.462	(.417, .506)	<.001
Employment duration (years worked)	.031	(.011, .051)	.003	.049	(.023, .074)	<.001	.019	(.010, .027)	<.001
Annual household income (log-transformed)	.233	(.115, .350)	<.001	.151	(.014, .289)	.031	.455	(.346, .563)	<.001
Body mass index	.041	(.007, .076)	.019	.037	(.015, .090)	.164	.050	(.033, .068)	<.001
Chronic health conditions	−.314	(−.485, −.143)	<.001	−.374	(−.596, −.152)	<.001	−.338	(−.411, −.264)	<.001
Slope	−.551	(−.800, −.302)	<.001	−.538	(−.738, −.338)	<.001	−.531	(−.622, −.441)	<.001
Employment duration* slope	.001	(−.005, .008)	.738	.000	(−.007, .007)	.942	.001	(−.002, .004)	.454

Asterisk denotes an interaction term.

In the full sample, women with some work experience (*M *= 12.41, *SD *= 2.84) compared to women with none (*M *= 10.08, *SD *= 3.73) had significantly higher levels of education, (*p *< .001, Cohen's *d *= −0.80). When excluding education as a covariate, having no formal work experience was significantly associated with lower TICS-27 scores at baseline (*p *< .001) and a greater rate of decline in cognitive performance over time (*p = .*04) compared to having one or more years of formal work experience. Compared to non-Latinx White women, non-Latinx Black and Latinx women performed lower at baseline (*p* < .001). When adjusting for education, differences between women with and without formal work experience were no longer significant, suggesting that disparities in educational attainment partially attenuated the relationship between formal work experience and cognitive decline. See Table 3 for full model details.

**Table 3:** Linear mixed effects models assessing the association of categorical employment duration, covariates, and cognitive decline assessed via TICS-27 total score in the full sample.

	Model 4a Full sample (without education as a covariate)			Model 4b Full sample (with education as a covariate)		
	B	CI	*p*	B	CI	*p*
Age (years)	−.229	(−.243, −.215)	<.001	−.214	(−.223, −.201)	<.001
Education (years)	–	–	–	.470	(.434, .505)	<.001
Race/Ethnicity						
White (ref)	–	–	–	–	–	–
Non−Latinx black	−2.873	(−3.143, −2.603)	<.001	−2.501	(−2.761, −2.240)	<.001
Latinx	−2.822	(−3.167, −2.476)	<.001	−1.004	(−1.374, −.635)	<.001
Annual household income (log-transformed)	.512	(.440, .583)	<.001	.356	(.278, .435)	<.001
Body mass index	.033	(.017,.049)	<.001	.050	(.034, .066)	<.001
Chronic health conditions	−.445	(−.514, −.376)	<.001	−.364	(−.431, −.297)	<.001
Slope	−.500	(−.538, −.463)	<.001	−.495	(−.533, −.457)	<.001
Work experience (yes/no)						
Yes (ref)	–	–	–	–	–	–
No	−1.290	(−1.80, −.774)	<.001	−1.005	(−2.096, .087)	.071
Work experience (yes/no) * slope						
Yes (ref)	–	–	–	–	–	–
No	−.172	(−.338, −.005)	.043	−.178	(−.532, .176)	.324

Asterisk denotes an interaction term. Ref: reference category.

When repeating the above analyses with the TICS-20 memory component subscore as the outcome variable, results largely remained the same. However, in the full sample, having no formal work experience was associated with lower performance on the TICS-20 memory component scores at baseline, and this effect persisted after adjusting for education (*p* = .04). See Tables 4, 5 for full details.

**Table 4:** Linear mixed effects models assessing the association of employment duration, covariates, and cognitive decline assessed via TICS−20 memory component subscore stratified by race and ethnicity.

	Model 2 Non-Latinx black			Model 3 Latinx			Model 1 Non-Latinx white		
	B	CI	*p*	B	CI	*p*	B	CI	*p*
Age (years)	−.164	(−.189, −.139)	<.001	−.144	(−.180, −.107)	<.001	−.183	(−.194, −.172)	<.001
Education (years)	.316	(.260, .372)	<.001	.137	(.083, .191)	<.001	.278	(.245, .312)	<.001
Employment duration (years worked)	.026	(.010, .042)	.001	.031	(.011, .051)	.002	.010	(.004, .017)	.002
Annual household income (log-transformed)	.111	(.027, .196)	.010	.090	(−.008, .187)	.073	.293	(.212, .376)	<.001
Body mass index	.016	(−.008, .040)	.197	.016	(−.023, .054)	.423	.031	(.018, .044)	<.001
Chronic health conditions	−.128	(−.248, −.007)	.038	−.232	(−.392, −.072)	.005	−.209	(−.265, −.153)	<.001
Slope	−.357	(−.569, −.145)	<.001	−.480	(−.655, −.305)	<.001	−.424	(−.501, −.346)	<.001
Employment duration * slope	−.001	(−.007, .004)	.624	.000	(−.006, .006)	.971	.001	(−.001, .003)	.304

Asterisk denotes an interaction term.

**Table 5:** Linear mixed effects models assessing the association of categorical employment duration, covariates, and cognitive decline assessed via TICS-20 memory component subscore in the full sample.

	Model 5a Full sample (without education as a covariate)			Model 5b Full sample (with education as a covariate)		
	B	CI	*p*	B	CI	*p*
Age (years)	−.190	(−.120, −.180)	<.001	−.183	(−.193, −.173)	<.001
Education (years)	–	–	–	.269	(.244, .294)	<.001
Race/Ethnicity						
White (ref)	–	–	–	–	–	–
Non-Latinx black	−1.467	(−1.66, −1.272)	<.001	−1.313	(−1.502, −1.125)	<.001
Latinx	−1.455	(−1.70, −1.205)	<.001	−.424	(−.683, −.165)	.001
Annual household income (log-transformed)	.305	(.253, .357)	<.001	.187	(.136, .238)	<.001
Body mass index	.019	(.007, .031)	.001	.026	(.015, .037)	<.001
Chronic health conditions	−.279	(−.329, −.229)	<.001	−.230	(−.278, −.182)	<.001
Slope	−.392	(−.424, −.360)	<.001	−.390	(−.422, −.358)	<.001
Work experience (yes/no)						
Yes (ref)	–	–	–	–	–	–
No	−.743	(−1.149, −.337)	<.001	−.421	(−.821, −.021))	.039
Work experience (yes/no) * slope						
Yes (ref)	–	–	–	–	–	–
No	−.124	(−.266, .018)	.086	−.106	(−.247, .036)	.143

Asterisk denotes an interaction term. Ref: reference category.

## Discussion

This study was primarily focused on elucidating the impact of formal work experience on the cognitive trajectories of older women (≥65), with a particular emphasis on historically marginalized groups, including non-Latinx Black, Latinx, and non-Latinx White women. We examined the impact of employment duration on cognitive decline over time, considering potential variations across different racial and ethnic backgrounds. Our results did not substantiate the first hypothesis. The number of years of employment did not affect the rate of cognitive decline, nor was there a differential impact by race and ethnicity among the women in our study. However, years worked did positively impact baseline performance, with each of the racial and ethnic groups having better TICS-27 and TICS-20 performance with greater employment history.

Discrimination based on race and ethnicity significantly influences employment opportunities and cognitive health trajectories. For non-Latinx Black Americans, the effects of institutional racism result in reduced access to high quality education, lower job attainment, and diminished income compared to other racial and ethnic groups (33). non-Latinx Black Americans disproportionately experience socioeconomic stressors, including financial precarity, lower-status jobs, periods of unemployment, inadequate housing, and the compounded challenges of food deserts and elevated crime rates (45). These factors hinder professional advancement and may negatively affect cognitive reserve. Additionally, it is well-known that chronic conditions may cluster among racial, ethnic, geographic, or cultural groups due to differences in health behaviors, access to healthcare, systemic inequities, and the numerous impacts of social determinants of health. Future work should consider whether specific chronic conditions uniquely affect the rate of cognitive decline in older women from different backgrounds and identities.

Educational attainment was included and excluded from certain models to determine whether the effect of years worked on cognitive performance was fully or partially explained by educational attainment. Given that educational attainment can confound the relationship between years worked and cognitive performance, it was essential to assess its attenuating effect. Although women without formal work experience performed worse at baseline and over time, these findings were attenuated by the inclusion of educational attainment. A larger sample would be needed to determine if educational attainment and formal work experience independently affect cognitive performance at baseline and over time or if these factors are interdependent.

We hypothesized that older women with no formal employment history would demonstrate a more pronounced rate of cognitive decline, supported by existing literature that highlights the role of job complexity in mitigating cognitive decline and reducing the risk of ADRD (22–28). Historically, women in the United States were restricted from fully participating in the formal workforce due to gender norms and legal discrimination until the Civil Rights Act of 1964. As a result, older cohorts of women (e.g., Silent Generation, Greatest Generation) often assumed demanding, unpaid roles as homemakers and caregivers, while their daughters (e.g., Baby Boomers) were encouraged to pursue “feminine” roles in the economy, such as teachers, nurses, administrative assistants, wait staff, and beauticians. These generational gender norms and legal segregation led to disparities in educational attainment, occupational status, and work complexity for women, which may have influenced their cognitive functioning and trajectory in later life (8, 42). This hypothesis aligns with the broader narrative that women are at an elevated risk for ADRD, a disparity that may be partly attributable to gender-based differences in CR stemming from historical discrimination in educational and occupational opportunities (5, 7, 8).

The Latinx cohort performed similarly at baseline to the non-Latinx Black cohort, despite having worked fewer years (21.36 vs. 32.68) and having lower educational attainment (8.26 vs. 11.72) on average. Age does not appear to be a confound, as these two groups were similar (74.20 vs. 74.31). If educational attainment was fully attenuating the relationship between formal work experience and cognitive performance, we might expect a significantly lower performance from our Latinx cohort. Because we do not see this (TICS-27: 11.81 vs. 11.91), it suggests that other factors such as education quality, cognitive reserve, test-wiseness, or a variety of other factors may be preserving our Latinx cohort's performance, at least on a univariate level.

Educational attainment is not solely a measure of knowledge or skill acquisition; it is often indicative of a broader spectrum of socioeconomic advantages. Higher education is correlated with higher-paying occupations, which may be a reflection of or a pathway to generational wealth. Such economic stability can afford an individual the luxury of engaging in health-promoting activities, accessing superior healthcare services, or reducing exposure to chronic social stressors—all of which are linked with preserving cognitive function (7, 16, 17). Moreover, prior literature has highlighted that education can enhance the brain's resilience to neuropathological damage by strengthening existing neural networks and facilitating the development of new ones (14). This reserve allows individuals to better cope with the structural changes associated with aging and potentially delays the onset of clinical manifestations of ADRD. However, older women with no formal work experience had fewer years of education on average in our study. It is possible that educational attainment and formal work experience impact cognitive health through similar processes for some women (e.g., cognitive reserve), but this is not likely to be true for all women. There may be a variety of reasons why a woman chooses to or is forced to abstain from the workforce (e.g., preference and economic ability to do so; health concerns; experiences of discrimination; gender roles and expectations), and each of these groups may have a different level of education and risk for cognitive decline in later life. Clearly, more work needs to be conducted along these lines.

Furthermore, despite our initial hypotheses, we did not observe an effect of employment duration on cognitive decline across non-Latinx Black, Latinx, or non-Latinx White older women. Several SDoH are known to impact cognitive health and risk of ADRD (46, 47), yet determinants especially prominent among historically underserved groups remain understudied (e.g., experiences of discrimination, exclusion from the formal workforce, nativity status; disparities in education quality) (34, 48). In our study, formal work experience affected cognitive performance and the rate of decline uniformly across non-Latinx Black, Latinx, or non-Latinx White women, which differs from the previous findings by Jester et al. (34) in which the number of years worked explained a sizeable proportion of the non-Latinx Black- non-Latinx White (6%) and Latinx- non-Latinx White (10%) disparities in cognitive performance at baseline. It is important to consider the possibility that a SDoH like work experience may influence performance at baseline, but not necessarily differences in longitudinal trajectories. Alternatively, it may be that when measuring cognitive trajectories by race and ethnicity, education simply explains more of the variance, thus attenuating the relationship. Although our findings paint an unclear image of the relationship between the number of years worked and cognitive decline across racial and ethnic groups, it highlights the need for ongoing research into the SDoHs that impact cognitive health.

Our findings are subject to methodological limitations, primarily stemming from the structure and availability of variables within the HRS. The HRS's classification of racial and ethnic groups is limited, which may not capture the full diversity within each category. It does not include individuals who do not fit into the predefined classifications or have missing race and ethnicity data. This categorization limitation could lead to a lack of representation and an incomplete understanding of the cognitive trajectories across a more diverse population. Furthermore, the variables available in the HRS allowed us to consider the quantity of education, but not the quality. The quality of education is a critical factor that can influence cognitive outcomes and varies based on several factors, such as socioeconomic status, school resources, and geographic location. The absence of data on the quality of education means that our analysis may only partially reflect the nuanced ways in which educational experiences impact cognitive health. Unfortunately, the reason for being absent from the formal workplace was not registered in the HRS dataset. Work is needed to understand how education and formal work experience influence each other, and how/whether they explain the same or different aspects of cognitive performance and cognitive decline. Furthermore, residual confounding may exist in our models despite our attempts at controlling for a variety of demographic, social, and health factors. Finally, this study's outcome variable was limited to the TICS-27. While TICS-27 is a widely used and validated measure for assessing global cognitive performance in the HRS, it does not capture the various domains of cognition in sufficient detail. Given that our sensitivity analysis utilizing the TICS-20 memory component subscore yielded slightly differing results, future work should include a full cognitive battery when possible.

In addition to its limitations, this study has several strengths. One of the main strengths is the substantial sample size of older women, which allows for more robust and generalizable findings. The richness of the HRS data is another significant strength, providing comprehensive information on a wide range of variables, including demographic, socioeconomic, and health-related factors. Furthermore, the longitudinal design of the HRS allows for examining changes over time, adding depth to our understanding of cognitive decline.

In conclusion, the relationship between formal work experience, education, race and ethnicity, and cognitive decline in older women is complex and multifaceted. Our study adds to the growing body of literature recognizing educational disparities’ crucial role in cognitive health outcomes. While our study points to educational disparities as a significant factor in cognitive decline, we recognize that education is intertwined with broader social issues, including gendered expectations and roles. The historical underrepresentation of women in higher education and the workforce, often due to caregiving responsibilities or cultural norms, especially in developing countries, merits further investigation to determine its impact on cognitive health. Moreover, gender discrimination and societal expectations have differentially shaped the educational and occupational opportunities available to women. As such, future studies should examine how these gendered experiences intersect with race and occupation to influence cognitive outcomes.

## Data Availability

Publicly available datasets were analyzed in this study. This data can be found here: https://hrsdata.isr.umich.edu/data-products.
